# Somatic Integration of Single Ion Channel Responses of α7 Nicotinic Acetylcholine Receptors Enhanced by PNU-120596

**DOI:** 10.1371/journal.pone.0032951

**Published:** 2012-03-30

**Authors:** Victor V. Uteshev

**Affiliations:** Department of Pharmacology , Southern Illinois University School of Medicine, Springfield, Illinois, United States of America; Virginia Commonwealth University, United States of America

## Abstract

Positive allosteric modulators of highly Ca^2+^-permeable α7 nicotinic acetylcholine receptors, such as PNU-120596, may become useful therapeutic tools supporting neuronal survival and function. However, despite promising results, the initial optimism has been tempered by the concerns for cytotoxicity. The same concentration of a given nicotinic agent can be neuroprotective, ineffective or neurotoxic due to differences in the expression of α7 receptors and susceptibility to Ca^2+^ influx among various subtypes of neurons. Resolution of these concerns may require an ability to reliably detect, evaluate and optimize the extent of α7 somatic ionic influx, a key determinant of the likelihood of neuronal survival and function. In the presence of PNU-120596 and physiological choline (∼10 µM), the activity of individual α7 channels can be detected in whole-cell recordings as step-like current/voltage deviations. However, the extent of α7 somatic influx remains elusive because the activity of individual α7 channels may not be integrated across the entire soma, instead affecting only specific subdomains located in the channel vicinity. Such a compartmentalization may obstruct detection and integration of α7 currents, causing an underestimation of α7 activity. By contrast, if step-like α7 currents are integrated across the soma, then a reliable quantification of α7 influx in whole-cell recordings is possible and could provide a rational basis for optimization of conditions that support survival of α7-expressing neurons. This approach can be used to directly correlate α7 single-channel activity to neuronal function. In this study, somatic dual-patch recordings were conducted using large hypothalamic and hippocampal neurons in acute coronal rat brain slices. The results demonstrate that the membrane electrotonic properties do not impede somatic signaling, allowing reliable estimates of somatic ionic and Ca^2+^ influx through α7 channels, while the somatic space-clamp error is minimal (∼0.01 mV/µm). These research efforts could benefit optimization of potential α7-PAM-based therapies.

## Introduction

Deficits in activation of highly Ca^2+^-permeable α7 nicotinic acetylcholine receptors (nAChRs) are associated with schizophrenia, Alzheimer's disease, aging and brain trauma, while enhancing this activation with nicotinic agonists may be therapeutic. Type-II positive allosteric modulators (α7-PAMs) of α7 nAChRs, such as PNU-120596, may become useful therapeutic tools supporting neuronal survival and function by enhancing deficient activation of α7 nAChRs [1], [2], [3], [4], [5]. Type-II α7-PAMs do not activate α7 nAChRs in the absence of nicotinic agonists, but increase the responsiveness of α7 nAChRs to nicotinic agonists by reducing the receptor desensitization [1], [2], [3], [4], [5], producing behavioral improvements in *in vivo* models [1], [3]. Specifically, PNU-120596 prolongs openings of α7 nAChR ion channels without producing significant changes in ion channel selectivity, single channel conductance, or Ca^2+^ permeability [1].

A moderate, persistent activation of α7 nAChRs can be neuroprotective [3], [6], [7], [8], [9] and modeled *ex vivo* using low concentrations of nicotinic agonists (e.g., physiological choline; ∼5–10 µM) enhanced by PNU-120596 [4], [5] and possibly, other Type-II α7-PAMs [2], [3]. Under these experimental conditions, detection of individual α7 channel openings in whole-cell experiments becomes possible in both voltage- and current-clamp configurations [4], [5]. By contrast, in the absence of PNU-120596, openings of individual α7 channels cannot be distinguished from noise in whole-cell recordings and thus, the extent of α7-mediated ionic and Ca^2+^ influx cannot be reliably detected and quantified. Therefore, PNU-120596 and possibly other α7-PAMs may be effective for enhancing and optimizing the potency of nicotinic agonists to a degree that permits low concentrations of nicotinic agonists such as physiological concentrations of choline to produce moderate, persistent activation of α7 nAChRs – effects that may support neuroprotection and cognitive performance *in vivo*. However, despite promising results, the initial optimism for potential therapeutic applications of PNU-120596 has been tempered by the concerns for cytotoxicity [9], [10]. In general, the same concentration of a given nicotinic agent can be neuroprotective, ineffective or neurotoxic due to differences in the expression density of functional α7 receptors and susceptibility to Ca^2+^ influx among various subtypes of neurons. The resolution of these concerns and ambiguities may require an ability to reliably detect, evaluate, manipulate and optimize the extent of α7-mediated somatic ionic and Ca^2+^ influx, a key determinant of the likelihood of neuronal survival and function.

However, while PNU-120596 allows detection of individual α7 channel activity in whole-cell patch-clamp experiments, it remains unclear whether activation of each individual α7 channel impacts the entire soma or only specific subdomains located in the channel/electrode vicinity, reflecting somatic electrotonic properties. Such a somatic electrical compartmentalization may reduce neuronal excitability and obstruct integration and detection of ion channel activity across the neuronal soma, causing an underestimation of whole-cell α7-mediated persistent ionic influx. A reliable quantification of individual α7 channel activity in whole-cell requires the neuronal soma to act as an effective integrator, allowing an individual α7 channel to depolarize the entire soma and thus, be reliably detected across the entire soma as well as by a patch-clamp electrode regardless of the electrode location on the somatic membrane. The degree of compliance to this requirement in central neurons is currently unknown. This study tests the hypothesis that the electrotonic properties of large hypothalamic tuberomammillary (TM) neurons (>20 µm) and hippocampal CA1 interneurons (>15 µm) do not impede somatic signaling allowing reliable estimates of the net charge generated by individual α7 ion channels in whole-cell recordings in the presence of PNU-120596. These results may benefit the search for an optimum in α7-mediated Ca^2+^ influx, neuronal excitability and survivability.

## Materials and Methods

### Animals

Young adult male Sprague-Dawley rats (P22-30) were used in accordance with the Guide for the Care and Use of Laboratory Animals (NIH 865-23, Bethesda, MD), and experimental protocols were approved by the Animal Care and Use Committee of Southern Illinois University School of Medicine.

### Tissue Preparation

Coronal whole-brain slices of 250-µm thickness containing hypothalamic TM nuclei and/or hippocampal CA1 region were cut in a sucrose-rich solution at 3°C using Vibratome-1000+ slicer (Leica Microsystems, Wetzlar, Germany). The sucrose-rich solution was of the following composition (in mM): sucrose 250, KCl 3, NaH_2_PO_4_ 1.23, MgCl_2_ 5, CaCl_2_ 0.5, NaHCO_3_ 26, glucose 10 (pH = 7.4, when bubbled with carbogen: 95% O_2_ and 5% CO_2_). Slices were then transferred to a temporary storage chamber where they were maintained for ∼30 min at 30°C in an oxygenated artificial cerebrospinal fluid (ACSF) of the following composition (in mM): NaCl 125, KCl 3, NaH_2_PO_4_ 1.23, MgCl_2_ 1, CaCl_2_ 2, NaHCO_3_ 26, glucose 10 (pH = 7.4, when bubbled with carbogen). Slices were then transferred to the long-term storage chamber and maintained at room temperature for up to 6 h bubbled with carbogen.

### Dual-patch recordings from a single cell in brain slices

The recording chamber was perfused with oxygenated ACSF at a rate of 1 ml/min using a perfusion pump (Model73160-20, Cole-Parmer, Vernon Hills, IL). TM neurons of the posterior hypothalamus and interneurons of the CA1 region of the hippocampus in brain slices were visually selected for electrophysiological recordings using an Olympus BX-51WI microscope (Olympus Inc, Center Valley, PA). Dual patch-clamp electrophysiological recordings were conducted using a MultiClamp-700B amplifier equipped with Digidata-1440A A/D converter (Molecular Devices, Sunnyvale, CA). Voltage and current traces were recorded in 20–100 s sweeps. No delays were allowed between sweeps. Data were filtered at 2–4 kHz, sampled at 10 kHz and stored on a personal computer. During analysis, data were filtered at 0.4–1 kHz for illustrations and comparison of traces. Patch pipettes were pulled using a Sutter P-97 horizontal puller (Sutter Instruments, Novato, CA). The pipette resistance was 4–6 MΩ when filled with the internal solution. In each experiment, a single neuronal soma was clamped with two patch electrodes: electrodes were advanced towards the opposite sides of the recorded soma under visual control and a cell-attached patch-clamp configuration with a gigaseal resistance was established for each electrode. The patch configuration of electrodes was later changed to whole-cell voltage- or current-clamp, as needed. The access resistances (<30 MΩ) were not compensated. Choline (5–10 µM) and PNU-120596 (1 µM) were present in ACSF during, and at least 40 min before, each experiment. The extracellular recording solution was identical to the ACSF used for the tissue preparation. The patch electrode solution contained (in mM): K-gluconate 140, NaCl 1, MgCl_2_ 2, Mg-ATP 2, Na-GTP 0.3, HEPES 10, KOH 0.42 (pH 7.4). Membrane voltages were not corrected for the liquid junction potential: V_LJ_(K-gluconate) = 16.2 mV.

### The lack of effects of R_f_

In four experiments with hypothalamic TM neurons, the feedback resistance (R_f_) of one or both of the recording amplifiers were switched from its whole-cell default value of 500 MΩ to 50 GΩ and step-like α7-mediated currents were recorded from the same cell by both electrodes in voltage-clamp. There were no visible differences between 500 MΩ and 50 GΩ traces (not shown) and the patterns of α7-mediated single channel openings recorded by the two electrodes remained identical: 100% of events detected by 50 GΩ amplifier were also detected by 500 MΩ amplifier and vice versa (not shown). Therefore, in whole-cell mode, the default feedback resistance (500 MΩ) provided a sufficient sensitivity for detecting as many α7 single channel events as does 50 GΩ feedback resistance and thus, reliable recordings of individual α7 channel activity in whole-cell did not require increasing R_f_.

### Drugs

In this study, 1 µM PNU-120596 was used. This concentration lies near the EC50 for potentiating effects of PNU-120596 in heterologous systems (EC50∼1.5 µM) (Grønlien et al., 2007; Young et al., 2008). The intravenous administration of 1 mg/kg PNU-120596 has been shown to elevate the concentration of PNU-120596 in the brains of rats to similar values (∼1.5 µM) (Hurst et al., 2005). PNU-120596 was obtained from the National Institute of Drug Addiction (NIDA) through the NIDA Research Resources Drug Supply Program or purchased from Tocris Bioscience (Ellisville, MO). Methyllycaconitine citrate (MLA) was purchased from Ascent Scientific Ltd. (Bristol, UK). Other chemicals were purchased from Sigma-Aldrich (St. Louis, MO). Choline-containing solutions were freshly made prior to each experiment from a 1 M stock which was kept at −20°C.

### Analysis

Only current-clamp data were used for automated analysis of ion channel activity (hypothalamic TM neurons, n = 16 and hippocamal CA1 interneurons, n = 8) using Clampfit-10 software, however all available data were visually analyzed in both current- and voltage-clamp modes. Both visual and automated analyses resulted in identical conclusions for both types of neurons tested. For automated analysis, two approaches were used. The first approach evaluated the standard deviation (S.D.) of the original and subtracted voltage traces: voltage traces recorded by electrode #2 were subtracted from traces recorded by electrode #1 using “Arithmetic” command (Clampfit-10) and then the values of S.D. of the original and subtracted traces were obtained using “Statistics” command (Clampfit-10). The second approach evaluated cross-correlations of the original voltage traces, estimated the correlation coefficient (R) and the probability that the estimated values of R were obtained by chance (i.e., p-value). The cross-correlation of traces was evaluated using “Cross-correlation” command with a ±10 ms lag. The value of R corresponding to 0 ms lag was chosen for statistical analysis and the t-value was determined from R as previously described [11]. The p-value was then calculated using its definition [11] and online resources provided by Dr. Richard Lowry (Vassar College, Poughkeepsie, NY; http://faculty.vassar.edu/lowry/rsig.html. To solve mathematical equations, Mathematica-2.2.3 software package (Wolfram Research, Inc. Champaign, IL) was used. To conduct one-way ANOVA test and the Bonferrani post-test, OriginPro-8 software package (Northampton, MA) was used. The results are presented as means±S.D.

## Results

### Hypothalamic TM neurons

**Figure 1:**
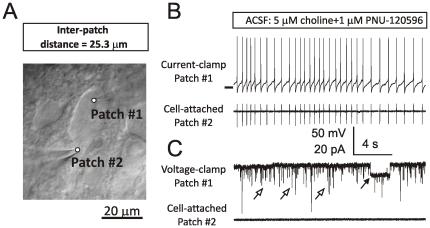
Reliability of physiological responses. A large hypothalamic TM neuron with two attached patch-clamp electrodes and an inter-patch distance of 25.3 µm (**A**). Examples of patch-clamp recordings obtained from the neuron shown in (**A**) under two experimental conditions: (**B**) Electrode #1 is in current-clamp (B, top trace), while Electrode #2 is cell-attached (B, bottom trace); and (**C**) Electrode #1 is in voltage-clamp at −70 mV (C, top trace), while Electrode #2 is cell-attached (C, bottom trace). Open arrows in (C) point at spontaneous synaptic currents. Closed arrow points at a step-like deviation corresponding to an individual α7 nAChR-mediated ion channel opening. ACSF always contained 5 µM choline plus 1 µM PNU-120596. A horizontal bar in (**B**) top trace indicates the membrane voltage of −50 mV. Currents were not injected in current-clamp.

**Figure 2:**
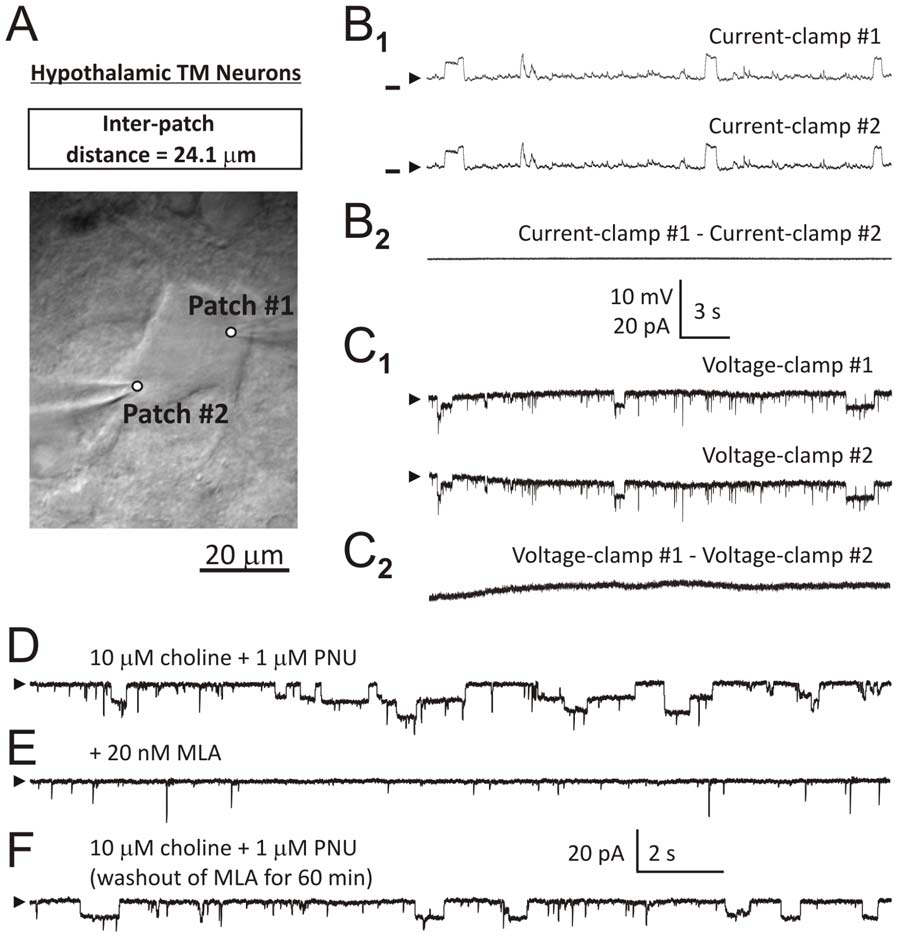
Somatic integration of individual α7 channel activity in hypothalamic TM neurons. A large hypothalamic TM neuron with two attached patch-clamp electrodes and an inter-patch distance of 24.1 µm (**A**). Examples of patch-clamp recordings obtained from the neuron shown in (**A**) when both patch electrodes are in current-clamp (**B**) or voltage-clamp at −70 mV (**C**). The bottom traces in B–C) are results of subtraction of trace #2 from trace #1 indicating identical patterns of TM α7 single ion channel activity recorded by the two electrodes (see text). Horizontal bars in (**B**) indicate the membrane voltage of −75 mV. A continuous hyperpolarizing current (50–120 pA) was injected into cells to cease spontaneous firing. The baselines are indicated by arrowheads. Step-like responses (**D**) were completely (**E**) and reversibly (**F**) blocked by 20 nM MLA, a selective α7 nAChR antagonist, added to ACSF.

Large histaminergic TM neurons were identified in coronal hypothalamic slices on the basis of their size and morphology, location within the slice, and the expression of high densities of functional α7 nAChRs [12] as well as strong I_h_ and I_A_ currents [13]. To activate α7 nAChRs, hypothalamic slices were perfused with ACSF containing 5 µM choline and 1 µM PNU-120596. In two experiments, 10 µM choline was used. To detect α7 single-channel events in whole-cell current-clamp experiments, spontaneous firing was inhibited by keeping the membrane voltage near −70 mV by injecting continuous hyperpolarizing currents (50–120 pA). The inter-patch distances were made as large as possible and images of the recorded TM neurons were taken during each experiment. The inter-patch distances were then measured off-line (Figures 1A and 2A, open circles). The average inter-patch distance was 21.0±2.9 µm (n = 16).

#### Reliability of physiological responses

The reliability of data obtained from the two recording electrodes was tested in each experiment: one of the electrodes was transitioned into current-clamp (Figure 1B, top trace) or voltage-clamp (Figure 1C, top trace), while the other electrode remained cell-attached (Figure 1B, C, bottom traces). Action potentials recorded by the electrode held in current-clamp (Figure 1B, top trace) were detected as synchronous membrane capacitance transients by the electrode held cell-attached (Figure 1B, bottom trace). By contrast, current deviations corresponding to α7 single channel openings (Figure 1C, close arrow) and spontaneous synaptic activity (Figure 1C, open arrows) recorded by the electrode held in voltage-clamp were undetected by the electrode held cell-attached (Figure 1C, bottom trace). These data demonstrate that the two recording electrodes did not cross-talk and voltage/current events recorded by one electrode were not relayed to another electrode by any means except directly from the recorded neuron.

#### Somatic integration of individual α7 channel activity in hypothalamic TM neurons

In each experiment, up to five 100 s long episodic traces were recorded in current- and voltage-clamp. In all TM neurons investigated in this study, the two patch electrodes recorded identical patterns (i.e., no mismatch) of α7 single channel events in both current-clamp (n = 14) and voltage-clamp (n = 16). Typical examples of current- and voltage-clamp traces are shown in Figure 2B_1_ and Figure 2C_1_, respectively. This conclusion was initially made on the basis of visual analysis and later confirmed by automated analysis using Clampfit-10 (see Methods). For automated analysis, two approaches were used. The first approach evaluated the standard deviation (S.D.) of the original (Figure 2B_1_) and subtracted voltage traces (Figure 2B_2_). The second approach evaluated cross-correlations between the original voltage traces recorded by the two electrodes and estimated the coefficient of correlation (R) and the probability that the estimated values of R are obtained by chance (i.e., p-value).

Step-like deviations recorded in hypothalamic TM neurons were completely and reversibly blocked by 20 nM methyllycaconitine (MLA), a selective antagonist of α7 nAChRs (n = 4, Figure 2D, E, F).

#### Analysis of standard deviations

Subtraction of traces recorded by electrode #2 (Figures 2B_1_ and 2C_1_, bottom traces) from the corresponding traces recorded by electrode #1 (Figures 2B_1_ and 2C_1_, top traces) eliminated all events in both current-clamp (Figure 2B_2_) and voltage-clamp (Figure 2C_2_) supporting the high degree of event correlation in traces recorded by the two electrodes. The values of S.D. of each of the original voltage traces obtained from the two electrodes (SD_1_ and SD_2_, Figure 2B_1_) were measured and compared to the S.D. of a trace obtained by subtraction of the original two voltage traces from one another (SD_1–2_, Figure 2B_2_). This analysis gave the following values: (n = 14): SD_1_ = 2.23±0.96 mV, SD_2_ = 2.27±0.98 mV and SD_1–2_ = 0.19±0.21 mV. Therefore, subtraction of original traces from one another resulted in a >10-fold drop in the S.D. compared to the S.D. of original traces (compare Figures 2B_1_ and 2B_2_). These results were consistent with the results of visual analysis indicating a lack of mismatch in the patterns of single α7 ion channel openings observed in dual-patch current-clamp (n = 14, Figure 2B_1_) and voltage-clamp (n = 16, Figure 2C_1_) experiments. To evaluate differences in the values of SD_1_, SD_2_ and SD_3_, a one-way ANOVA test and the Bonferrani post-test were used. The values of SDs were found to be significantly different (F(2,39) = 31.16, p<0.0001). The Bonferonni post-test determined that differences between SD_1_ and SD_2_ were statistically insignificant (p>0.05), while differences between SD_1_ and SD_1–2_ as well as SD_2_ and SD_1–2_ were statistically significant (p<0.05).

#### Analysis of cross-correlations

**Figure 3:**
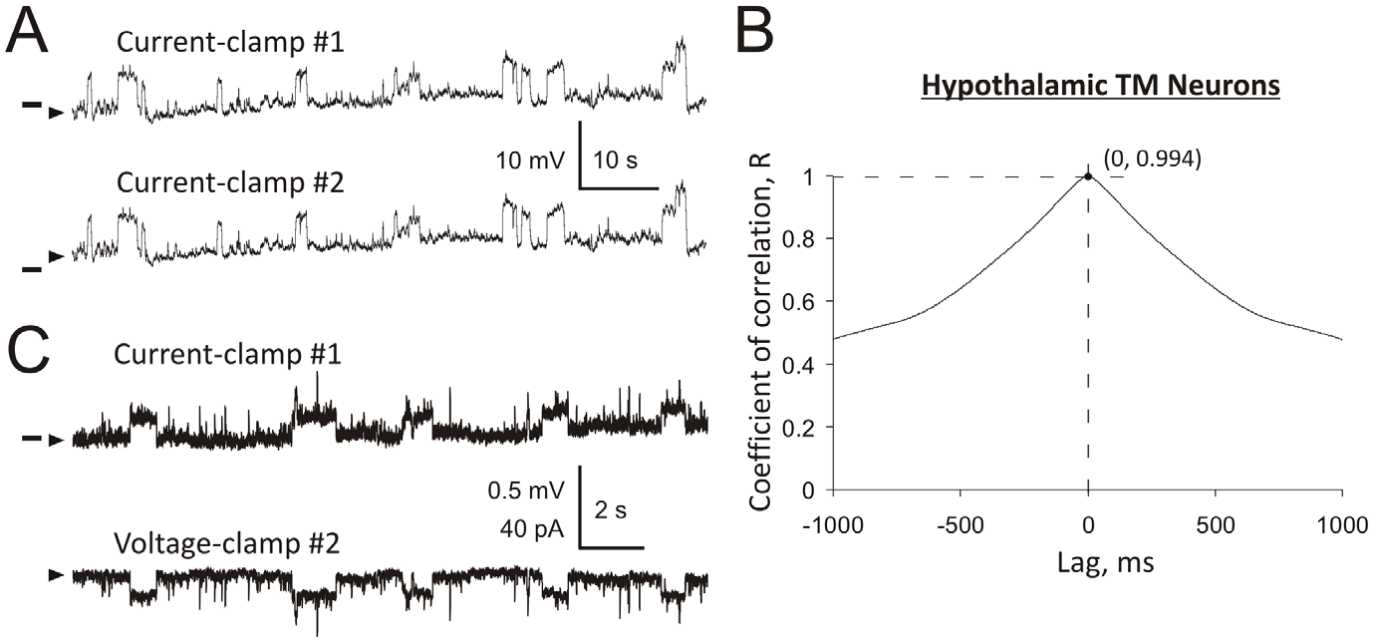
Analysis of cross-correlations and space-clamp in hypothalamic TM neurons. An example of traces recorded by two patch-clamp electrodes in current-clamp (**A**). Results of cross-correlation analysis (**B**). The correlation coefficient at lag = 0 ms (R = 0.9993) was used to estimate the significance of cross-correlation between the two traces (see Methods). Results obtained in the same experiment when electrode #2 was switched to voltage-clamp at −70 mV (**C**). Electrode #1 remained in current-clamp and detected small (∼0.22 mV) space-clamp errors (C, top trace) in the quality of voltage-clamp provided by electrode #2. Horizontal bars indicate the membrane voltage of −70 mV (**A**) and −70.2 mV (**C**). A continuous hyperpolarizing current (50–120 pA) was injected into cells to cease spontaneous firing (**A**). The baselines are indicated by arrowheads.

Episodic (100 s long) voltage traces recorded from the same TM neuron by two patch electrodes were analyzed to determine the degree of identity (i.e., cross-correlation) among individual α7 ion channel events. As a result, the correlation coefficient was determined for each pair of voltage traces. Figures 3A, B illustrate typical examples of whole-cell voltage traces recorded from a TM neuron by two patch electrodes (Figure 3A) and the values of correlation coefficient as a function of time lag (in ms) obtained in the same experiment (Figure 3B). The correlation coefficient corresponding to a 0 ms lag was then used for estimation of the t- and p-values (see Methods). In the example shown in Figure 3B, the correlation coefficient R = 0.9993 corresponding to lag = 0 ms is marked by a small dot at the intersection of dashed lines. The mean correlation coefficient evaluated over all current-clamp experiments was R = 0.996±0.005 (n = 14) indicating an extremely strong correlation between pairs of voltage traces (Figure 3A). This correlation was found to be highly significant (p<0.0001, n = 14, see Methods).

#### Equipotential somatic space in hypothalamic TM neurons

To evaluate potential errors in clamping the membrane voltages (i.e., space-clamp) related to large (∼20 µm) inter-patch distances (Figures 1A and 2A), in five experiments, one of the two electrodes was always held in current-clamp, while the second electrode switched between voltage- and current-clamp modes. In these experiments, the average inter-patch distance was 21.70±3.76 µm (n = 5). When the second electrode was held in voltage-clamp (Figure 3C, bottom trace), the mean amplitude of step-like voltage deviations recorded by the first electrode held in current-clamp was 0.22±0.10 mV (n = 5, Figure 3C, top trace) and thus, of the same magnitude as SD_1–2_. By contrast, when both electrodes were held in current-clamp, the mean amplitude of step-like voltage deviations was 2.87±0.64 mV (n = 5, Figure 3A), i.e., a >10-fold greater value. These results demonstrate that in TM neurons, the somatic space-clamp error is small (<10%) and develops as a slow function of the somatic distance (∼0.01 mV/µm). These data present the TM somatic space as nearly equipotential.

### Hippocampal CA1 interneurons

**Figure 4:**
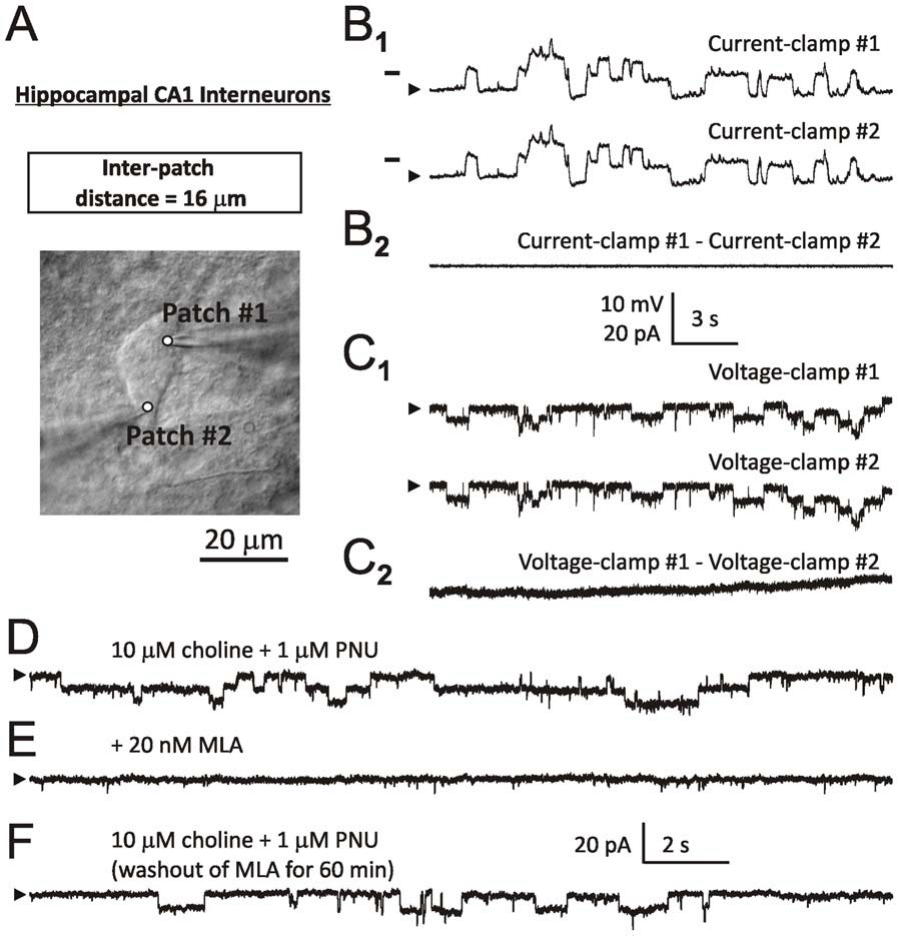
Somatic integration of individual α7 channel activity in hippocampal CA1 interneurons. A hippocampal CA1 interneuron with two attached patch-clamp electrodes and an inter-patch distance of 16 µm (**A**). Examples of patch-clamp recordings obtained from the neuron shown in (**A**) when both patch electrodes are in current-clamp (**B_1_**) or voltage-clamp at −80 mV (**C_1_**). Results of subtraction of trace #2 from trace #1 in current-clamp (**B_2_**) and voltage-clamp (**C_2_**) indicating identical patterns of α7 single ion channel activity recorded by the two electrodes (see text). Horizontal bars in (**B**) indicate the membrane voltage of −75 mV. A continuous hyperpolarizing current (50–120 pA) was injected into cells to cease spontaneous firing. The baselines are indicated by arrowheads. Step-like responses (**D**) were completely (**E**) and reversibly (**F**) blocked by 20 nM MLA added to ACSF.

To determine whether the observed effects are pertinent to other α7 nAChR-expressing neurons, the identical dual-patch experimental protocol was applied to hippocampal CA1 interneurons expressing functional α7 nAChRs [5], [14] with single-channel activity observable in whole-cell patch-clamp experiments [5]. The results obtained from hippocampal CA1 interneurons and hypothalamic TM neurons were similar. Interneurons were identified on the basis of their morphology and location within the CA1 *Stratum Radiatum* region of the hippocampus. To activate α7 nAChRs, hippocampal slices were perfused with ACSF containing 5 µM choline and 1 µM PNU-120596. In four experiments, 10 µM choline was used. To detect α7 single-channel events in whole-cell current-clamp experiments, occasional spontaneous action potentials were prevented by keeping the membrane voltage near or below −70 mV by injecting continuous hyperpolarizing currents (50–150 pA). Similar to the case of hypothalamic TM neurons, the inter-patch distances were made as large as possible to introduce the largest electrical resistance between the two patches. However, hippocampal CA1 interneurons were generally smaller than TM neurons and therefore, the average inter-patch distance for interneurons was shorter. Images of the recorded hippocampal CA1 interneurons were taken during each experiment and the inter-patch distances were measured (Figures 4A, open circles). The average inter-patch distance was 14.7±1.6 µm (n = 8).

#### Somatic integration of individual α7 channel activity in hippocampal CA1 interneurons

On the basis of visual analysis, in all eight hippocampal CA1 interneurons tested in this study, the two patch electrodes recorded identical patterns of α7 single channel events in both current-clamp (n = 8) and voltage-clamp (n = 6). Typical examples of current- and voltage-clamp traces are shown in Figure 4B and Figure 4C, respectively. This conclusion was later confirmed by automated analysis using Clampfit-10 (see description of experiments with TM neurons above). Specifically, the analysis of Standard Deviations gave the following values: (n = 8): SD_1_ = 1.35±0.83 mV, SD_2_ = 1.36±0.85 mV and SD_1–2_ = 0.11±0.06 mV. These results were similar to those obtained using hypothalamic TM neurons and demonstrated that subtraction of original traces from one another resulted in a >10-fold drop in the S.D. To evaluate differences in the values of SD_1_, SD_2_ and SD_3_, a one-way ANOVA test and the Bonferrani post-test were used. The values of SDs were found to be significantly different (F(2,21) = 8.79, p<0.01). The Bonferonni post-test determined that differences between SD_1_ and SD_2_ were statistically insignificant (p>0.05), while differences between SD_1_ and SD_1–2_ as well as SD_2_ and SD_1–2_ were statistically significant (p<0.05). As in the case of hypothalamic TM neurons, step-like deviations recorded in hippocampal CA1 interneurons were completely and reversibly blocked by 20 nM MLA (n = 4, Figure 4D, E, F).

**Figure 5:**
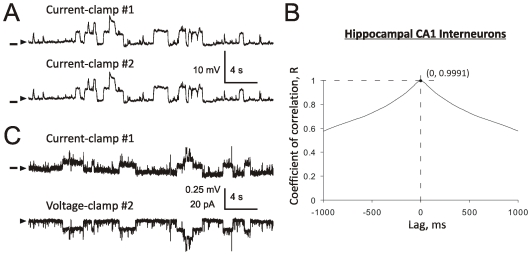
Analysis of cross-correlations and space-clamp in hippocampal CA1 interneurons. An example of traces recorded by two patch-clamp electrodes in current-clamp (**A**). Results of cross-correlation analysis (**B**). The correlation coefficient at lag = 0 ms (R = 0.9991) was used to estimate the significance of cross-correlation between the two traces (see Methods). Results obtained in the same experiment when electrode #2 was switched to voltage-clamp at −80 mV (**C**). Electrode #1 remained in current-clamp and detected small (∼0.12 mV) space-clamp errors (C, top trace) in the quality of voltage-clamp provided by electrode #2. Horizontal bars indicate the membrane voltage of −79 mV (**A**) and −79.4 mV (**C**). A continuous hyperpolarizing current (50–120 pA) was injected into cells to cease spontaneous firing (**A**). The baselines are indicated by arrowheads.

Figures 5A, B illustrate examples of typical voltage traces recorded from a hippocampal CA1 interneuron by two patch electrodes (Figure 5A) and the value of correlation coefficient as a function of time lag (in ms) obtained in the same experiment (Figure 5B). In this example, the correlation coefficient R = 0.9991 corresponding to lag = 0 ms is marked by a small dot at the intersection of dashed lines (Figure 5B). Analysis of cross-correlations among events recorded by the two patch electrode indicated an extremely strong correlation between pairs of voltage traces with the mean correlation coefficient, <R> = 0.992±0.009 (n = 8). This correlation was found to be highly significant (p<0.0001, n = 8).

#### Equipotential somatic space in hippocampal CA1 interneurons

Similar to the case of hypothalamic TM neurons, the space-clamp error in hippocampal CA1 interneurons was found to be small (<10%). When both electrodes were held in current-clamp (Figure 5A), the mean amplitude of step-like voltage deviations in interneurons was 2.46±1.05 mV (n = 5). By contrast, when one of the electrodes was held in current-clamp (Figure 5C, top trace) and the second electrode was held in voltage-clamp (Figure 5C, bottom trace), the mean amplitude of voltage deviations in interneurons was 0.12±0.07 mV (n = 5) and thus, similar to the magnitude of SD_1–2_. In these experiments, the average inter-patch distance was 14.2±1.6 µm (n = 5). These results demonstrate that as in the case of hypothalamic TM neurons, in hippocampal CA1 interneurons the somatic space-clamp develops as a slow function of the somatic distance (∼0.01 mV/µm). Therefore, the somatic space of hippocampal CA1 interneurons also appears to be nearly equipotential.

## Discussion

A moderate persistent activity of highly Ca^2+^-permeable α7 nAChRs can be neuroprotective [3], [6], [7], [8], [9] and may be achieved by endogenous levels of choline enhanced by α7-PAMs (e.g., PNU-120596) [9]. These conditions can be modeled in whole-cell current- and voltage-clamp experiments in brain slices, while quantification of α7-mediated persistent ionic influx integrated by neuronal somata could provide a rational basis for optimization of the levels of α7 nAChR activation that promotes neuronal survival and function. Likewise, the results of this study may help to define conditions that reduce cytotoxicity, a key concern in the use of Type-II α7 PAMs, because these compounds robustly enhance α7 activation and Ca^2+^ influx [10].

In the presence of physiological choline (∼5–10 µM) and 1–2 µM PNU-120596 in ACSF, persistent openings of individual α7 ion channels can be detected in whole-cell patch-clamp experiments in hypothalamic and hippocampal slices [4], [5]. Under these and similar conditions, the extent of α7-mediated persistent ionic influx may act as a key determinant of the likelihood of neuronal survival and function [10]. However, quantification of this influx remained elusive because activation of individual α7 channels may not be integrated across the neuronal soma and reliably detected in whole-cell patch-clamp experiments. A reliable quantification of individual α7 channel activity in whole-cell recordings requires neuronal soma to act as an effective integrator, allowing an individual α7 channel to depolarize the entire soma and thus, be detected by a patch-clamp electrode regardless of the electrode location on the somatic membrane. By contrast, somatic electric compartmentalization may obstruct somatic integration and detection of α7-mediated step-like voltage/current deviations and lead to errors in estimation of α7-mediated somatic ionic influx and net charge recorded in whole-cell experiments in the presence of nicotinic agonists and α7-PAMs. It would also make the patterns of α7 single-channel events depend on the location of recording electrode on the neuronal surface. Such a dependence was not observed in this study, as in all sixteen experiments with hypothalamic TM neurons and eight experiments with hippocampal CA1 interneurons, the patterns of α7 ion channel activity recorded from a single neuron by two electrodes were identical (Figures 2B, C, 3A, B, C, 4B, C and 5A, B, C). Moreover, the results of simultaneous voltage- and current-clamp recordings from a single neuron (Figure 3C and 5C) revealed that the somatic space-clamp error in both TM neurons and CA1 interneurons is minimal (<10%) and the accumulation of voltage error was a slow function of somatic distance (∼0.01 mV/µm). Therefore, these results do not support the presence of somatic compartmentalization in hypothalamic TM neurons and hippocampal CA1 interneurons and confirm the hypothesis that, in the presence of PNU-120596 and possibly other α7-PAMs, activation of an individual α7 ion channel depolarizes the entire soma and is detected and integrated across the entire soma.

These conclusions are supported by previous observations that the amplitudes of α7-mediated step-like currents in voltage-clamp (*i∼4–5 pA*) and voltages in current-clamp (*V∼8–10 mV*) in TM neurons comply with the Ohm's law (*i = V/r*), where the values of *r* fall near the values of TM input resistance (∼*500 MΩ*) [4]. Therefore, α7 single-channel currents elicit voltage deviations that recharge the majority of the somatic membrane and not a small segment of it. In the latter case, the value of *r* would have been proportionally smaller, indicating the presence of electrical compartmentalization in the vicinity of the recording electrode. Furthermore, in experiments with hippocampal CA1 pyramidal neurons in the presence of 2 µM PNU-120596 and 10 µM choline, all recorded action potentials were generated by clearly detected α7-mediated step-like depolarizations [5]. These observations argue against the presence of single α7 channel activity undetected in whole-cell recordings because such an undetected activity would have produced randomly occurring action potentials not associated with α7-mediated step-like depolarizations clearly observed in these current-clamp recordings. The present study directly demonstrates that in the presence of PNU-120596, the patterns of activity of individual α7 nAChRs as seen by the soma are independent of the somatic position of electrodes and therefore, despite their large sizes, somata of hypothalamic TM neurons (>20 µm) and hippocampal CA1 interneurons (>14 µm) act as nearly equipotential volumes and effective signal integrators. This conclusion justifies and allows evaluation of α7-mediated persistent ionic and Ca^2+^ influx integrated by somata of hypothalamic TM neurons and hippocampal CA1 interneurons under various experimental conditions [4]. Similar conclusions may apply to other central neurons.

Although it appears beneficial for neurons to express functional α7 nAChRs, as moderate activation of these highly Ca^2+^-permeable receptor-channels can be therapeutic [1], [3], [6], [7], [8], inadequate (i.e., deficient or extensive) activation of α7 nAChRs may cause neuronal dysfunction, damage and death. Specifically, a prolonged activation of α7 nAChRs by endogenous choline and/or ACh in the presence of α7-PAMs may be cytotoxic and may disrupt neuronal circuitry. Therefore, the search for an optimum in activation of α7 nAChRs and Ca^2+^ entry is critical in designing therapeutic approaches aimed at supporting neuronal survival and function [9]. The results of this study suggest that the rate of α7-mediated persistent ionic influx and its Ca^2+^ component integrated by the soma can be reliably estimated in conventional whole-cell recordings in the presence of PNU-120596 in hypothalamic TM neurons and hippocampal CA1 interneurons because electrotonic properties of these cells do not impede somatic signaling. These, results may help to reveal and quantify conditions that promote neuroprotection. Once the rate of α7 persistent ionic influx is determined, a correlation between this rate and the level of neuroprotection can be established and optimized for various subtypes of α7-expressing neurons.

Taken together, this and previous studies demonstrated that in the presence of physiological choline (i.e., 5–10 µM) and 1–2 µM PNU-120596, patch-clamp whole-cell experiments can provide reliable estimates of the number of simultaneously open α7 channels (N_total_P_open_∼0.27), α7-mediated persistent ionic influx (∼1.4 pA) and Ca^2+^ influx (∼0.14 pA) integrated by the TM soma [4]. Similar estimates performed in hippocampal CA1 pyramidal neurons [5] indicated a >10-fold weaker expression of functional pyramidal α7 nAChRs compared to hippocampal CA1 interneurons and the TM. The ability to evaluate and manipulate the level of persistent activation of α7 nAChRs by physiological choline in the presence of α7-PAMs may reveal important relationships between the extent of α7-mediated somatic persistent ionic influx and neuronal survival and function in the search for a Ca^2+^ optimum [9]. By contrast, in the absence of PNU-120596, openings of individual α7 channels cannot be distinguished from noise in whole-cell recordings and thus, cannot be reliably detected, quantified or optimized. Therefore, these observations suggest an intriguing possibility of establishing and optimizing a quantitative relationship between the level of neuroprotection by nicotinic agonists in the presence of Type-II α7-PAMs and the extent of α7-mediated somatic persistent ionic and Ca^2+^ influx evaluated within the relevant *ex vivo* models.
